# Death Following the Administration of Ether, Etc.

**Published:** 1876-05

**Authors:** E. L. Holmes

**Affiliations:** Professor of Diseases of the Eye and Ear, Rush Medical College; Chicago


### DEATH FOLLOWING THE ADMINISTRATION OF ETHER, AT THE ILLINOIS CHARITABLE EYE AND EAR INFIRMARY.

By E. L. HOLMES, M.D.,

Professor of Diseases of the Eye and Ear, Rush Medical College.

(Read before the Chicago Medical Society, April 3, 1876.)

Mr. M., in the 74th year of his age, came under my care for the extraction of cataract. Unfortunately, there was chronic conjunctivitis in each eye, which foreshadowed a protracted treatment. The patient was exceedingly obese, especially in the abdominal region—had a remarkably large and finely developed head, with a short and very thick neck. The upper and lower limbs were peculiarly deficient in development, apparently from early childhood, for they were relatively small and extremely short; the hands and feet were also uncommonly small. While the body was very nearly normal in length, the limbs were so short that the whole height of the patient was but four feet three inches. His weight was 184 pounds.

The general health had always been quite good, enabling the patient to perform his duties in an important State office, which he had held nearly forty years, till he became blind. The patient had become slightly asthmatic, and was troubled with some cough. Although faint rales in the chest could be heard, the action of the heart and lungs presented no symptom indicative of failing health. I doubt whether the pulse could be called weak. The appetite, digestion and excretory functions were quite normal. There was, however, a tendency to undue perspiration in exercising, or when the temperature of his room was a little elevated.

In the latter part of December, the eyes were in so favorable a condition that I considered it safe to perform a preliminary iridectomy. The patient took no food after breakfast. Squibb's stronger ether, turned upon a large porous towel, folded several times and covered with paper, was given to the patient, while lying on his back with the head slightly elevated. The patient breathed very quietly, till he seemed to be unconscious, when coughing commenced. This was no more violent than is often observed when this anæsthetic is administered. Scarcely any mucus or saliva collected in the throat. Soon after this, the breathing ceased, and the face became remarkably livid, while the pulse continued regular and strong. Pressure on the chest aroused the respiration, when more ether was given and the operation on each eye completed without further unpleasant symptoms. There was no vomiting.

The wounds in the corneal border healed readily, although from a repeated recurrence of slight conjunctivitis I did not consider the eyes to be in a condition fit for the extraction of cataract for about three months.

Meanwhile the general health had continued as good in every respect as it had been. Dr. I. N. Danforth had given the patient simple remedies to relieve a trifling cough and asthma, and to overcome the undue perspiration. The asthma never prevented sleep in the recumbent position. At half past three, on the 27th of March, Squibb's ether was again administered under precisely the same circumstances as at the previous operation. I must confess that nothing in the patient's condition excited the least suspicion, either in Dr. Danforth or myself, that a fatal result could follow. The thought never entered my mind, although I had some solicitude regarding the success of an extraction of cataract in an eye which might be considered predisposed to inflammation. The patient was in a cheerful state of mind, and inhaled the ether quietly till about half a pound had been consumed, when quite violent coughing commenced. This was soon followed by an extremely livid appearance of the face and then by a cessation of breathing. I placed my finger on the pulse, found it failing in strength and directed my assistant to remove the towel, and others in the room to raise the foot of the bed as high as possible. Meantime I raised the tongue, and with the other hand made very forcible pressure every few seconds on the side of the chest.

Respiration was at once re-established, as also full action of the heart, when I directed the foot of the bed to be let down. The lividity in a great measure disappeared. Even at this moment I did not think of danger, but, without giving more ether, rapidly commenced and completed the section, and, as the iridectomy had already been performed, as rapidly removed the lens. This passed through the wound at once with only the least possible manipulation. The bandage was speedily adjusted. I cannot think two minutes elapsed from the commencement of the section to the final adjustment of the bandage. I made the steps as rapidly as was consistent with care, being not quite certain that the patient had taken ether enough to keep him unconscious sufficiently long for the possible delay in "delivering" the lens, which occasionally happens. My usual method of applying the compressive bandage reduces the time to a minimum, since I place an elastic band around the forehead ready in any emergency to be slipped over the eyes. I am confident, therefore, that all this required less than two minutes, when I observed that the patient did not breathe, that the face was more livid and the pulse very weak.

The same course was pursued as before the operation as regards drawing up the tongue, raising the foot of the bed and inducing artificial respiration, and, in addition, by forcing air into the lungs (?) by the breath. At the end of a minute or more, I could feel no pulse nor hear the heart; the face had become exceedingly livid.

The efforts I have described, except raising the foot of the bed and forcing the breath into the mouth were continued an hour and a quarter.

There were present my assistant, the matron, the resident pupil, and one of the nurses. Galvanism was not employed. No autopsy was obtained.

In neither case was the towel held so close to the face as to prevent the entrance of sufficient air into the lungs.

It is perhaps due to myself to state, that for nearly fourteen months in 1853–4, I was resident assistant in the Massachusetts General Hospital, at Boston, and administered anæsthetics, under the supervision of the surgeons, in a large number of cases. Subsequently I witnessed the administration of chloroform very frequently, in European hospitals. For about eleven years ending 1867, I used chloroform almost exclusively in hospital and private practice. In several instances in my practice the use of chloroform was followed by the most alarming symptoms; the action of the heart and lungs absolutely ceased, as far as could be observed by auscultation of the chest. In every instance there was sudden and apparently complete syncope. Fortunately no fatal result occurred.

After observing two or three of these cases and witnessing numerous instances in which danger seemed imminent, I made the experiment of placing the patient with head down on an inclined plane of at least 45°. I found without exception, in extreme cases and in cases where there was slight enfeeblement of the pulse with pallor of the face, that this change of position caused immediate fullness of the pulse and flushing of the cheeks. I was led to this step by the appearance of the patients, and by the recollection of seeing one of my early medical instructors seize a child, apparently dead from shock and loss of blood, by the feet and hold it a moment head downward. This brought to my mind the injunction of another teacher (in obstetrics), to raise the foot of the bed as high as possible in cases of dangerous hæmorrhage.

I experimented very many times, and soon resolved never to administer chloroform unless the patient was placed on a suitable board, so the position could be easily changed with the least delay.

Although I have long since abandoned the use of chloroform, I am fully convinced that this treatment—by increasing the hydrostatic pressure of the blood at the nervous centres—reduces the dangers from chloroform to a minimum, and at this minimum, chloroform can scarcely be considered as an especially dangerous anæsthetic. I apprehend, physicians in resorting to this method, too often fail to apply it *promptly*, and in such a manner that the whole body is on a very steep inclined plane. These points are of primary consequence as compared to elevating the tongue.

A notice of these observations was published in the transactions of the Illinois State Medical Society for the year 1868. This was copied in a large number of medical journals—as far as I know—without comment regarding originality. It was only till somewhat recently that I learned from Dr. J. Marion Sims in person, and from his published address, that the method had been employed years before by Nêlaton.

Ether, as well as chloroform, sometimes causes dangerous cessation of breathing. I believe this, with the secondary effect, lividity, is due to the action of the anæsthetic on the nervous centres of respiration. I had observed during my first experiments that patients in this state placed in the position above described invariably and speedily began to breathe. The elevated column of blood seemed to excite the nerve-centres.

I may add, that I have seen two cases in which the administration of ether was followed, as the patients regained consciousness, by most violent and protracted neuralgia of the left arm.

